# Concomitant Infection With Porcine circovirus 2 and Porcine Epidemic Diarrhea Virus Does Not Increase Enteritis Severity in Experimentally Infected Weaned Piglets

**DOI:** 10.1155/tbed/2698648

**Published:** 2026-04-25

**Authors:** Priscilla F. Gerber, Jianqiang Zhang, Andrew Noel, Bin Wang, Yaowei Huang, Patrick G. Halbur, Tanja Opriessnig

**Affiliations:** ^1^ Department of Infectious Diseases and Public Health, City University of Hong Kong, Kowloon Tong, Hong Kong, cityu.edu.hk; ^2^ Department of Veterinary Diagnostic and Production Animal Medicine, College of Veterinary Medicine, Iowa State University of Science and Technology, Ames, Iowa, USA; ^3^ Guangdong Laboratory for Lingnan Modern Agriculture, College of Veterinary Medicine, South China Agricultural University, Guangzhou, Guandong, China, scau.edu.cn; ^4^ Vaccines and Diagnostics Department, Moredun Research Institute, Penicuik, UK, moredun.org.uk

**Keywords:** antibody, coinfection, PCV2, PEDV, virus shedding

## Abstract

**Background:**

Young pigs are often coinfected with porcine circovirus 2 (PCV2) and porcine epidemic diarrhea virus (PEDV). This study aimed to determine the impact of coinfection of pigs with PCV2 and PEDV. Forty 16‐day‐old crossbred mixed‐sex piglets were assigned to four groups (*n* = 10/group, NEG‐CONTROL, PCV2‐CONTROL, PCV2+PEDV, and PEDV‐CONTROL). At day postinoculation (dpi) 0, NEG‐CONTROL pigs were inoculated with saline, PCV2‐CONTROL pigs were inoculated with PCV2, PCV2+PEDV pigs were inoculated with PCV2 and PEDV, and PEDV‐CONTROL pigs were inoculated with PEDV.

**Results:**

No clinical signs were observed in the NEG‐CONTROL and PCV2‐CONTROL group pigs throughout termination of the study at dpi 21. Other than mild to moderate diarrhea, which lasted for about 7 days, no other clinical signs associated with PEDV or PCV2 infection were observed in the PEDV‐CONTROL and PEDV+PCV2 groups.

**Conclusion:**

Coinfection of PEDV and PCV2 had no effect on virus shedding, serum antibody profile, and macroscopic or microscopic lesions.

## 1. Background

Circovirus porcine 2 (PCV2), one of the smallest viruses to infect mammals, can be found in nearly all pig farms, in both healthy and diseased pigs [1]. In affected pigs, PCV2 can cause a range of clinical manifestations, such as respiratory distress and diarrhea in pigs at any age or enteric disease characterized by diarrhea associated with granulomatous enteritis in grow‐finish pigs [2]. PCV2 infects immune cells, causing lymphocytic depletion in lymphoid tissues, and can enhance infection and replication of other pathogens under field conditions [1]. This leads to potentially increased severity of PCV2 diseases (PCVDs) as demonstrated with experimental coinfections with pathogens such as porcine reproductive and respiratory syndrome virus (PRRSV) and *Mycoplasma hyopneumoniae* [1].

Coinfection of PCV2 and porcine epidemic diarrhea virus (PEDV) has been described in pigs with diarrhea in commercial farms [3–7]. PEDV is an alphacoronavirus that causes acute diarrhea and occasional vomiting in pigs of all ages, with high mortality in naïve neonatal piglets that progressively decreases with pig age [8]. PEDV infects enterocytes mainly in the jejunum and ileum, leading to marked villus atrophy in the small intestine [9].

It has been shown that transplacental infection with PCV2 potentiates PEDV pathogenicity in 3‐day‐old piglets by causing more severe intestinal villous atrophy and an increased number of PEDV antigen‐positive cells in intestinal tissues compared to PEDV only‐infected pigs [10]. Although PCV2‐positive cells were found in the lamina propria of the jejunum and ileum, no lymphoid depletion, a hallmark of PCV2‐systemic infection, or increase in the number of PCV2‐infected cells in lymphoid tissues in pigs coinfected with PEDV was observed in that study [10]. These results indicate that transplacental PCV2 infection exacerbated PEDV‐induced disease and lesions. In addition, an in vitro study investigating the order of infection of PEDV and PCV2 in virus replication found that when the porcine ileum‐derived cell line IPI‐FX was first infected with PCV2, it inhibited subsequent PEDV replication, whereas when cells were simultaneously infected with PCV2 and PEDV, the replication of PEDV was enhanced [11]. Collectively, the in vivo and in vitro studies point to an interaction between these viruses.

This study aimed to investigate the interaction of PCV2 and PEDV coinfection in weaned piglets, which is commonly observed in the field. The piglets used in this study had PCV2 maternal antibodies to mimic field conditions, as most commercial sows have antibodies against PCV2 because of vaccination or natural infection [12, 13]. We hypothesize that concomitant infection with PEDV and PCV2 enhances PEDV replication in pigs, resulting in more severe enteric disease. We also investigated the effect of PEDV infection on the development of PCVD.

## 2. Methods

### 2.1. Animals, Housing, and Experimental Design

The experimental protocol in this study was approved by the Iowa State University Institutional Animal Care and Use Committee (Approval Number 5‐14‐7804‐S; approved on July 9, 2014). The experiment was conducted between July and August of 2014.

**Figure 1:**
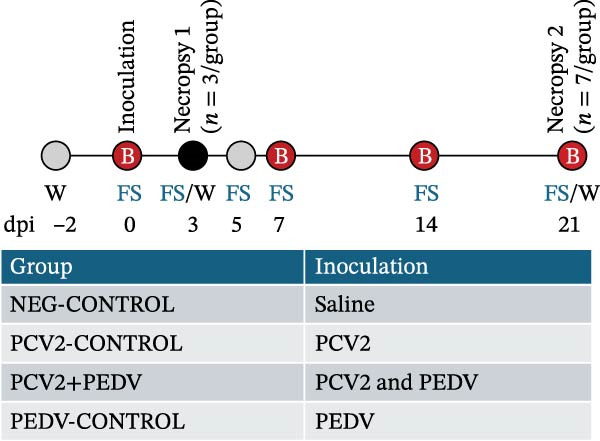
Experimental groups and timeline. B, blood collection; dpi, day postinoculation; FS, fecal swab collection; PCV2, porcine circovirus type 2; PEDV, porcine epidemic diarrhea virus; W, weight assessment.

The experimental design is shown in Figure 1. Forty 14‐day‐old crossbred mixed‐sex piglets were acquired from sows serologically negative for PEDV, PRRSV, porcine parvovirus (PPV), and swine influenza A virus (IAV) and seropositive for PCV2. Piglets were ear‐tagged, randomly assigned to four groups of 10 pigs each, and housed in four separate rooms at a biosafety level 2 research facility at Iowa State University, Ames, Iowa, USA. The random number generator in Excel (Microsoft Corporation) was used to assign pigs to groups and rooms based on ear tag numbers. Each room had 18 m^2^ of solid concrete floor space, separate ventilation systems, and one nipple drinker. Rubber or plastic floor toys for environmental enrichment were present in each pen. At 16 days of age, pigs were inoculated with PCV2, PEDV, or saline. All groups were fed ad libitum a balanced, pelleted, complete feed ration free of animal proteins (Heartland Coop, Prairie City, Iowa, USA). After inoculation, pigs were monitored daily for clinical signs, including diarrhea, lethargy, sneezing, and coughing. The experimental unit was a single animal.

Three randomly selected pigs in each group were euthanized by intravenous injection of pentobarbital sodium (Fatal Plus, Vortech, Dearborn, Michigan, USA) at a dose of 2 mL per 10 kg live weight at day postinoculation (dpi) 3, and the seven remaining animals in each group were euthanized in the same manner on dpi 21 (Figure 1). Blood samples were collected in serum separator tubes (Fisher Scientific, Pittsburgh, Pennsylvania, USA) at dpi 0, 7, 14, and 21. Serum was harvested following centrifugation at 3000 × g for 10 min at 4°C, and tested for anti‐PEDV and anti‐PCV2 IgG antibodies by enzyme‐linked immunosorbent assay (ELISA) and PCV2 DNA by quantitative (q) PCR. Fecal samples were collected with polyester swabs at dpi 0, 3, 5, 7, 14, and 21. Swabs were stored at −80°C in 5 mL plastic tubes containing 1 mL of sterile saline solution and tested for PEDV RNA by quantitative reverse transcriptase (RT)‐PCR. Pigs were weighed at dpi −2, 4, and 21.

### 2.2. Viral Inoculation

Pigs in the PCV2‐CONTROL and PEDV+PCV2 groups were experimentally infected with 5 mL of PCV2b isolate NC16845 [14] at a concentration of 10^4.2^ TCID_50_/mL by a combination of the intranasal route (slowly dripping 1.5 mL into each of the two nostrils) and the intramuscular route (intramuscular injection of 2 mL into the neck). PEDV‐CONTROL and PEDV+PCV2 pigs were experimentally infected with 10 mL of the 5th passage of the PEDV isolate 13‐19338E [15] at a tissue culture infective dose (TCID_50_) of 5 × 10^2^ per mL via the oral route. Similarly, NEG‐CONTROL pigs were sham‐inoculated orally with 10 mL of sterile cell culture media, 3 mL of sterile saline intranasally, and 2 mL intramuscularly. On inoculation and sampling days, treatments were handled in the following order: NEG‐CONTROL, PCV2‐CONTROL, PCV2+PEDV, PEDV‐CONTROL. Inoculum doses were chosen to cause moderate disease based on previous experiments using similar animal models.

### 2.3. Serology

Serum samples were tested for anti‐PCV2 antibodies using a PCV2 recombinant capsid protein‐based ELISA [16]. Samples with a sample‐to‐positive ratio (S/P) equal to or greater than 0.2 were considered positive. Serum samples were also tested for IgG anti‐PEDV antibodies using a PEDV recombinant spike 1 antigen‐based ELISA [17]. Samples with an OD value equal to or greater than 0.25 were considered positive.

### 2.4. Detection and Quantification of Viral Nucleic Acids

Serum samples were tested for PCV2 DNA by qPCR assay targeting a conserved region in ORF1 [14]. Fecal swab suspensions were tested for the presence of PEDV RNA by qRT‐PCR assay targeting a conserved region in the ORF1b gene [18]. To confirm that the viruses from the experimentally infected pigs were the same as the virus in the inoculum, the PCV2 ORF2 gene [14] and the PEDV S1 gene [18] from selected samples were amplified and sequenced.

### 2.5. Necropsy, Microscopic Examination, and Immunohistochemistry (IHC)

Randomly selected pigs were euthanized by intravenous pentobarbital sodium overdose (Fatal Plus, Vortech Pharmaceuticals, Ltd., Dearborn, MI, USA) and necropsied. The extent of macroscopic lung lesions ranging from 0% to 100% was estimated and blindly scored [19]. The size of superficial inguinal lymph nodes was scored [14]. Sections of lymph nodes (superficial inguinal, external iliac, mediastinal, tracheobronchial, and mesenteric), tonsil, spleen, kidney, liver, nine sections of small intestines (duodenum, jejunum, and ileum) and three sections of large intestines (colon) were collected at necropsy, fixed in 10% neutral‐buffered formalin, and routinely processed for histological examination. Microscopic lesions were blindly evaluated by a veterinary pathologist. Lymph nodes, spleen, and tonsil were evaluated for the presence and degree of lymphoid depletion and granulomatous replacement of follicles, ranging from 0 (normal) to 3 (severe) [14]. Lung sections were scored for the presence and severity of interstitial pneumonia, ranging from 0 (normal) to 6 (severe diffuse) [19]. Sections of small and large intestines were evaluated for the presence of inflammation, villus atrophy, and necrosis, ranging from 0 (normal) to 3 (severe) [14, 18, 20].

PCV2‐specific antigen was detected by IHC on lymph nodes, tonsil, and spleen using a polyclonal rabbit anti‐PCV2 serum [21]. PCV2 antigen scores ranged from 0 (no signal) to 3 (more than 50% of lymphoid follicles contained cells with PCV2 antigen staining) [14]. PEDV‐specific antigen was detected by IHC on selected formalin‐fixed and paraffin‐embedded sections of intestinal sections using a monoclonal antibody specific for PEDV (BioNote, Hwaseong‐si, Gyeonggi‐do, Korea) [22, 23]. PEDV antigen scores ranged from 0 (no signal) to 3 (more than 50% of villous enterocytes containing cells with PEDV staining) [18].

### 2.6. Statistical Analysis

Summary statistics from all groups were calculated to assess the overall quality of the data. Repeated data measurements (PCR and serology) were log‐transformed before analysis. Data for all animals were included in the analysis, that is, 10 animals per group for analyses up to dpi 3 and the 7 remaining animals per group for further analyses. Differences between piglets’ treatment groups at any given time for repeated measures were analyzed by repeated measures analysis of variance (PCR and serology), and nonrepeated measures (ADG, macroscopic, and microscopic lesions) were analyzed by analysis of variance. The significance of differences between more than two variables was tested using Tukey’s Honest Significant Difference test. Data were reported as least square means (LSM) and standard error of means (SEM). A *p* ≤ 0.05 was considered significant, and *p* ≤ 0.10 was considered a trend. Data were analyzed using JMP 13 statistical software (SAS Institute Inc., Cary, NC, USA).

## 3. Results

### 3.1. Clinical Signs

No clinical signs were observed in the NEG‐CONTROL and PCV2‐CONTROL group pigs. There was no detection of PEDV or PCV2 nucleic acid or antibodies in the NEG‐CONTROL pigs throughout the study, indicating that biocontainment was achieved during the experiment. In addition, pigs in the PCV2‐CONTROL group did not seroconvert to PEDV or shed PEDV RNA in feces, and PCV2 DNA was not detected in pigs in the PEDV‐CONTROL group. Sequencing of PEDV and PCV2 PCR‐positive samples confirmed 100% identity of the retrieved viruses compared to the strains used for inoculation.

The clinical presentation was similar in PEDV‐CONTROL and PEDV+PCV2 groups. Other than mild to moderate diarrhea, no other clinical signs associated with PEDV or PCV2 infection were observed in the PEDV‐CONTROL and PEDV+PCV2 groups. In the PEDV‐infected groups, pigs developed pasty to fluid diarrhea around dpi 3, which lasted until dpi 7–10 and returned to normal afterwards. From the time of inoculation to the termination of the study, there was no difference in the average daily weight gain (*p* = 0.54) among the four groups.

### 3.2. Anti‐PCV2 Antibody Detection

PEDV and PCV2 coinfection did not change serological and virus shedding profiles compared to singular PEDV and PCV2 infections. All pigs, regardless of the treatment group, had high levels of antibodies against PCV2 at dpi 0 (LSM S/P ratios ± SEM ranging from 0.82 ± 0.03 [PCV2+PEDV] to 0.67 ± 0.03 [PCV2‐CONTROL]) due to the presence of maternal antibodies (Figure 2). The PCV2 antibody levels remained high for the duration of the trial. No anamnestic response was observed after PCV2 inoculation in the PCV2‐CONTROL and PCV2+PEDV groups. The LSM antibody levels for the PCV2+PEDV group were higher at dpi 0 (0.82 ± 0.03) compared to antibody levels at dpi 21 (0.61 ± 0.04), indicating a decline in maternal antibody levels (*p* = 0.02). The difference in antibody levels of the remaining groups was not significant (*p*  > 0.10) (Figure 2).

Figure 2Serological response and virus detection are given as the least squares group mean (LSM) and standard errors. (A) Anti‐PCV2 IgG sample‐to‐positive (S/P) ratios. Pigs from all groups had PCV2 maternal antibodies at the beginning of the study. An S/P ratio equal to or greater than 0.2 was considered positive (dashed red line). (B) Anti‐PEDV IgG optical density (OD) values. An OD value equal to or greater than 0.25 was considered positive (dashed red line). (C) PCV2 log genomic copies in serum samples. (D) PEDV log genomic copies in fecal swabs. Pigs in the NEG‐CONTROL group were sham‐inoculated. Group denomination indicates the pathogen used to inoculate 16‐day‐old pigs within a group (PCV2‐CONTROL, PCV2+PEDV, and PEDV‐CONTROL).

**Figure:**
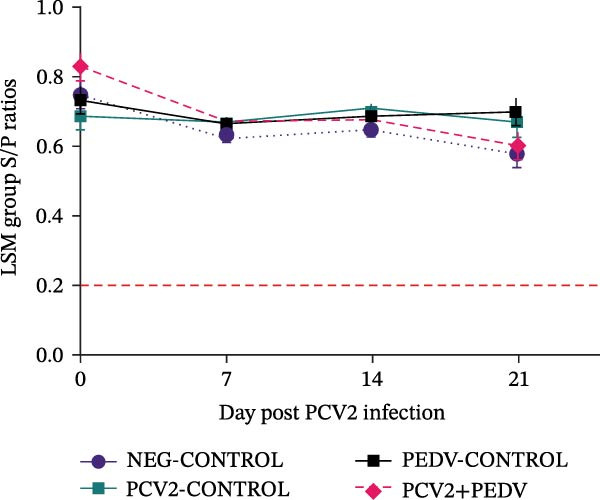
(A)

**Figure:**
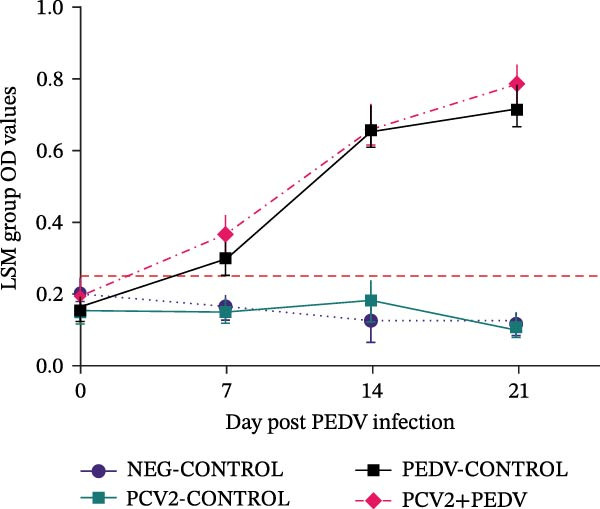
(B)

**Figure:**
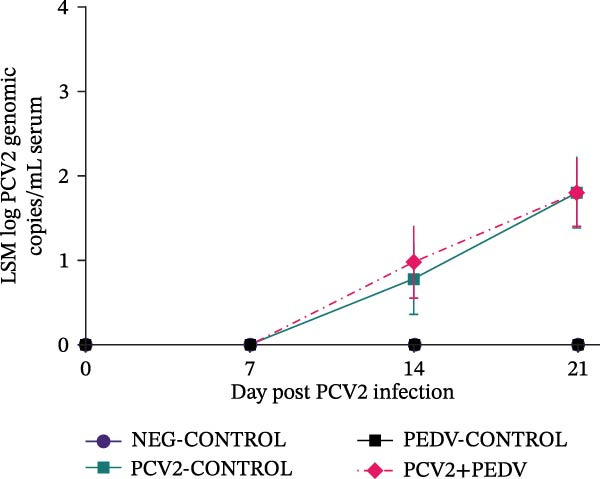
(C)

**Figure:**
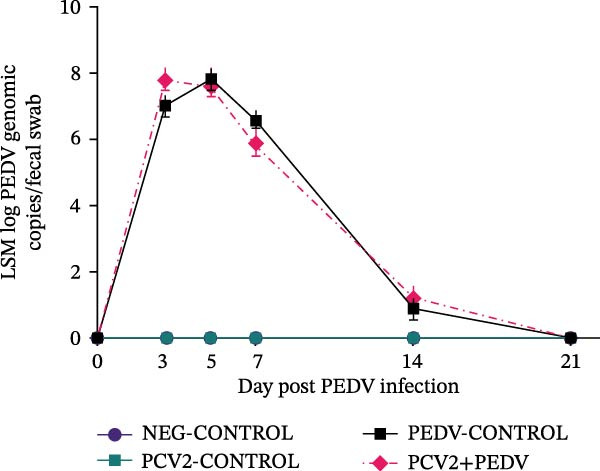
(D)

### 3.3. Anti‐PEDV Antibody Detection

Antibodies against PEDV were initially detected by dpi 7 in 42% (3/7) PEDV‐CONTROL pigs and 71% (5/7) PCV2+PEDV pigs. By dpi 14, all PEDV‐inoculated pigs had seroconverted (Figure 2). There was no difference in the LSM group antibodies levels between pigs in the PEDV‐CONTROL and PCV2+PEDV pigs.

### 3.4. PCV2 DNA Detection in Serum

PCV2 DNA was not detected in the NEG‐CONTROL and PEDV‐CONTROL pigs throughout the study. PCV2 DNA was only detected at dpi 14 and dpi 21 in 28.5% (2/7) PCV2‐CONTROL pigs and in 28.5% (2/7) PCV2+PEDV pigs. PCV2 load in positive serum samples ranged from 5.26 to 7.01 log genomic copies per mL. There was no difference in the LSM PCV2 log genomic copies in the serum of the two PCV2‐infected groups (Figure 2).

### 3.5. PEDV RNA Detection in Fecal Swabs

PEDV RNA was detected in all fecal swabs of PEDV‐infected groups at dpi 3, 5, and 7. By dpi 14, fewer pigs were shedding PEDV RNA in the PEDV (3/7) and PCV2+PEDV (2/7) groups. At dpi 21, none of the pigs were shedding PEDV RNA. There was no difference in the LSM PEDV log genomic copies in feces of the two PEDV‐infected groups (Figure 2).

### 3.6. Macroscopic Lesions, Microscopic Examination, and IHC

By dpi 4, gross lesions were limited to segmentally thin‐walled small intestines containing undigested feed and fluid gray–green intestinal contents in PEDV‐infected pigs. By dpi 21, no remarkable macroscopic lesions were observed except lymphadenopathy (score 2) in 1/7 PCV2‐CONTROL pigs, 3/7 PCV2+PEDV pigs, and 3/7 PEDV‐CONTROL pigs. Microscopic lesions and IHC staining are summarized in Table 1, and representative IHC staining sections are shown in Figure 3.

Figure 3Immunohistochemistry for PEDV (A–C) and PCV2 (D–F). (A) Small intestinal sections of a pig from the PEDV+PCV2 group, 3 days postinfection, showing high amounts of PEDV‐antigen (brown staining) in small intestinal villi with decreased villus to crypt ratio. (B) PEDV negative control. No apparent PEDV staining and normal villus to crypt ratio. (C) PEDV antigen positive control section with scattered brown staining. (D) Lymph node of pig from the PEDV+PCV2 group, 21 days postinfection, showing low‐to‐moderate amounts of PCV2‐antigen (brown staining predominantly in macrophages in a lymph follicle). (D) PCV2 negative control. No apparent PCV2 microscopic change and staining. (E) PCV2 antigen positive control section with scattered brown staining.

**Figure:**
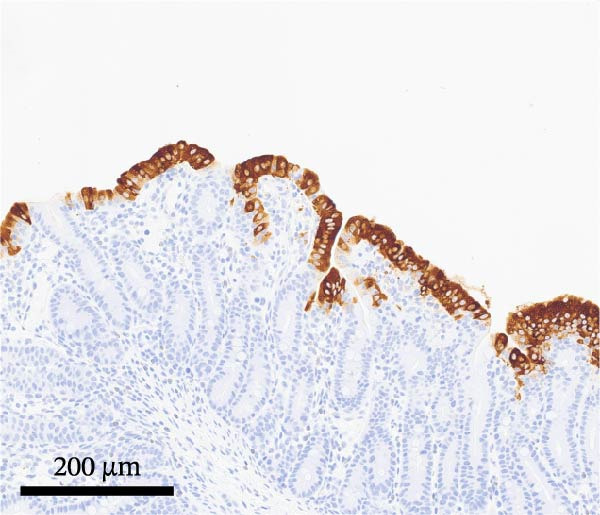
(A)

**Figure:**
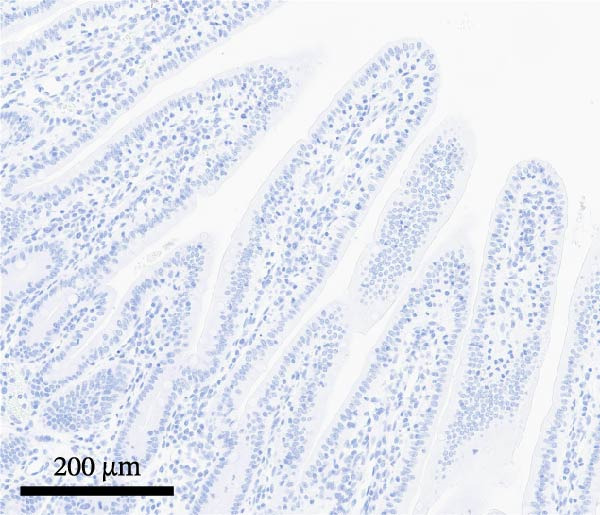
(B)

**Figure:**
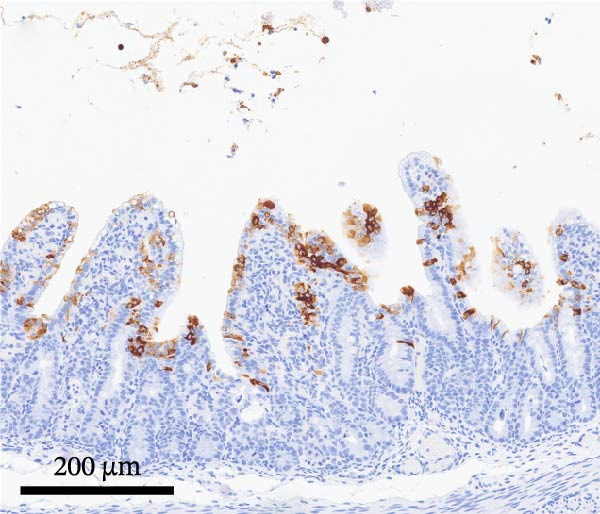
(C)

**Figure:**
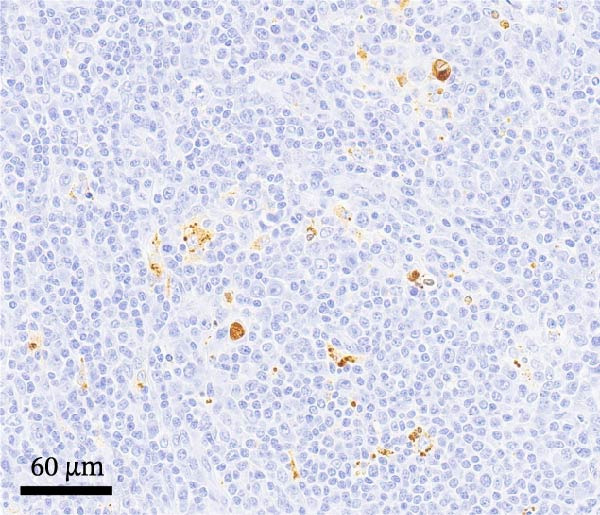
(D)

**Figure:**
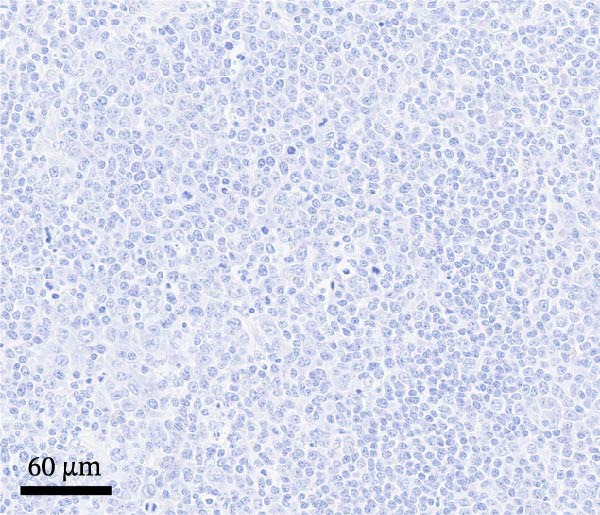
(E)

**Figure:**
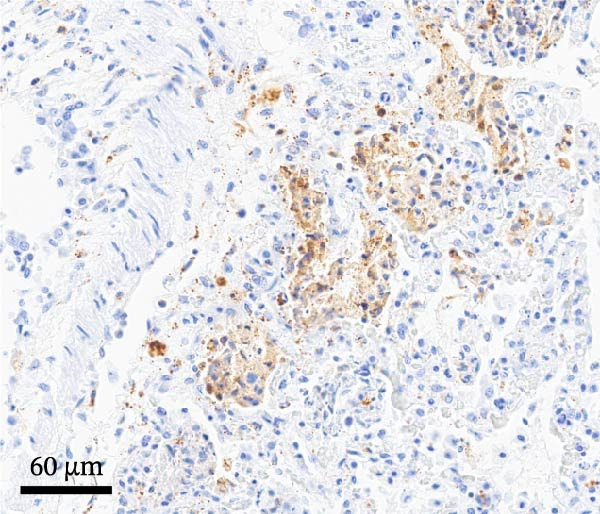
(F)

**Table 1:** Microscopic lesions and presence of PEDV or PCV2 staining determined by immunohistochemistry in small intestine and lymphoid organs (lymph nodes, spleen, and tonsils) at day postinoculation 3 or 21.

Group	dpi 3		dpi 21	
	Intestinal lesions^a^	PEDV IHC^b^	Lymphoid lesions^c^	PCV2 IHC^d^
NEG	0/3	0/3	0/7	0/7
PCV2	0/3	0/3	0/7	0/7
PEDV+PCV2	**3/3 (2,3)**	**3/3 (3,3)**	**2/7 (1,1)**	**1/7 (1,2)**
PEDV	**3/3 (2,3)**	**3/3 (3,3)**	0/7	0/7

*Note:* Data presented as prevalence (median positive score, highest score in the group). No lymphoid lesions or PCV2 IHC staining were recorded on dpi 3 in any group. No small intestine lesions or PEDV IHC staining were recorded on dpi 21 in any group. Bold values indicate that at least one animal was positive in that category.

^a^Microscopic lesions included villous atrophy and lymphocytic inflammation in small intestine section and were scored as 0 = absent to 3 = severe.

^b^Prevalence and amount of PEDV antigen in small intestine sections were scored as 0 = absent to 3 = abundant.

^c^Microscopic lesions included lymphoid depletion and granulomatous replacement of follicles and were scored as 0 = absent to 3 = severe.

^d^Prevalence and amount of PCV2 antigen were scored as 0 = absent to 3 = abundant for each tissue (lymph nodes, spleen, and tonsils).

## 4. Discussion

This study investigated the interaction of PCV2 and PEDV coinfection in weaned 16‐day‐old piglets with high maternal antibody levels against PCV2. The results did not support our hypothesis that PCV2 infection would potentiate PEDV enteritis in pigs, as evidenced by no increase in severity of clinical signs, or macroscopic or microscopic lesions in the coinfected group compared to single infection with PEDV for the duration of the study. We also did not observe an apparent potentiating effect of PEDV in PCV2 replication and manifestation of PCVD under the conditions of this study.

The lack of additive effects of PCV2 and PEDV coinfection in the severity of disease observed in this study contrasts with a previous study showing increased PEDV pathogenicity in neonatal piglets transplacentally infected with PCV2 [10]. Several reasons could explain the difference in outcomes, including the order of pathogen infection, the age of pigs at infection, and the virus strains and dose used. In the current study, only 2/7 pigs exhibited mild lymphoid lesions at dpi 21, in addition to the low PCV2 DNA load in serum (~2 log PCV2 genomic copies per mL serum), which suggests that the PCV2 infection was not effective, and this may also have contributed to the lack of additive effects observed. The mild PCV2 infection may have resulted from the presence of PCV2‐specific passive antibodies and the relatively low PCV2 inoculum dose used in the current study. Future studies should also consider using higher inoculum doses to better reproduce disease.

The order of infection has been shown to be important in disease expression in experimental PCV2 coinfection models. For example, in a PRRSV/PCV2 coinfection model in 5‐week‐old pigs, PCV2 infection 1 week before infection with a high pathogenic PRRSV strain reduced PRRSV pathogenicity by partially suppressing PRRSV‐associated cytokine storm compared to PRRSV single infection or PRRSV and PCV2 concurrent infection [24]. The order of infection also affects PCVD expression. In a recent study, PCVD was induced when pigs were inoculated with PRRSV 2 weeks before PCV2 or with concurrent inoculation of PCV2 and PRRSV but not when PCV2 infection occurred 2 weeks before PRRSV inoculation [25]. Further research on the sequence of infections of PEDV and PCV2 and their effects on virus–host interaction would be useful.

Previous studies investigating coinfection in PEDV‐related watery diarrhea cases from commercial pig farms found PCV2 and PEDV concurrent infection rates of 32.7% (35/107) in South Korea and 57.8% (44/76) in China [3, 4]. In both studies, the clinical samples were from neonatal piglets. Age‐dependent clinical manifestation of PEDV has been extensively demonstrated, with neonatal piglets being more susceptible to infection and developing more severe disease compared to weaned pigs [9, 26]. The moderate PEDV‐induced diarrhea in the presence of severe atrophic enteritis is expected in pigs infected at this age group [20]. PCV2 may potentiate PEDV infection in an age‐dependent manner, with increased disease severity when neonatal infection occurs. The lack of interaction between viruses could also be related to the presence of maternal antibodies against PCV2. Although maternally derived antibodies did not preclude PCV2 infection in this study, they could have reduced the likelihood of PCVD manifestation [13] and changes in PCV2+PEDV interactions in vivo.

## 5. Conclusion

Under the conditions of this study, we observed no exacerbation of PEDV or PCV2 pathogenicity in concurrently infected weaned piglets as measured by clinical signs, virus shedding, and macroscopic and microscopic lesions compared to single infection with either pathogen. Further studies using piglets without maternal antibodies against PCV2 and different sequences of pathogen infection are required.

## Author Contributions

Priscilla F. Gerber, Jianqiang Zhang, Tanja Opriessnig, and Patrick G. Halbur contributed to the animal trial. Priscilla F. Gerber, Jianqiang Zhang, Bin Wang, Yaowei Huang, Tanja Opriessnig, and Patrick G. Halbur contributed to the methodology and analyses of data. Priscilla F. Gerber prepared Figures 1 and 2. Tanja Opriessnig prepared Table 1. Patrick G. Halbur and Andrew Noel prepared Figure 3. Priscilla F. Gerber wrote the main manuscript.

## Funding

This study was not funded by an outside source.

## Disclosure

All authors reviewed and approved the final manuscript.

## Ethics Statement

The experimental protocol in this study was approved by the Iowa State University Institutional Animal Care and Use Committee (Approval Number 5‐14‐7804‐S; approved on July 9, 2014).

## Consent

The authors have nothing to report.

## Conflicts of Interest

The authors declare no conflicts of interest.

## Data Availability

All data generated during this study are included in this published article.
